# Structure-driven CO_2_ selectivity and gas capacity of ionic clathrate hydrates

**DOI:** 10.1038/s41598-017-17375-1

**Published:** 2017-12-08

**Authors:** Hidenori Hashimoto, Tsutomu Yamaguchi, Hiroyuki Ozeki, Sanehiro Muromachi

**Affiliations:** 1 Graduate School of Environmental Science, Toho University, 2-2-1 Miyama, Funabashi-shi, Chiba, 274-8510 Japan; 2 Research Institute of Energy Frontier (RIEF), National Institute of Advanced Industrial Science and Technology (AIST), 16-1 Onogawa, Tsukuba, Ibaraki, 305-8569 Japan

## Abstract

Ionic clathrate hydrates can selectively capture small gas molecules such as CO_2_, N_2_, CH_4_ and H_2_. We investigated CO_2_ + N_2_ mixed gas separation properties of ionic clathrate hydrates formed with tetra-*n*-butylammonium bromide (TBAB), tetra-*n*-butylammonium chloride (TBAC), tetra-*n*-butylphosphonium bromide (TBPB) and tetra-*n*-butylphosphonium chloride (TBPC). The results showed that CO_2_ selectivity of TBAC hydrates was remarkably higher than those of the other hydrates despite less gas capacity of TBAC hydrates. The TBAB hydrates also showed irregularly high CO_2_ selectivity at a low pressure. X-ray diffraction and Raman spectroscopic analyses clarified that TBAC stably formed the tetragonal hydrate structure, and TBPB and TBPC formed the orthorhombic hydrate structure. The TBAB hydrates showed polymorphic phases which may consist of the both orthorhombic and tetragonal hydrate structures. These results showed that the tetragonal hydrate captured CO_2_ more efficiently than the orthorhombic hydrate, while the orthorhombic hydrate has the largest gas capacity among the basic four structures of ionic clathrate hydrates. The present study suggests new potential for improving gas capacity and selectivity of ionic clathrate hydrates by choosing suitable ionic guest substances for guest gas components.

## Introduction

CO_2_ capture technologies in industry are necessary to be developed to reduce the vast CO_2_ emission^1–3^. Gas separation by ionic clathrate hydrates is promising due to their unique gas selectivity and low operation pressure^4–7^. As well as gas hydrates which are widely known for natural methane hydrates ionic clathrate hydrates are also investigated for their applications such as cold energy storage^8–11^, gas storage^12–15^ and gas separation^4,16,17^. Potential applications are based on unique thermodynamic properties of ionic clathrate hydrates, i.e., greatly moderated formation pressure and temperature compared to those for gas hydrates: <1 MPa for ionic clathrate hydrates^18,19^ and 3 MPa for structure I type gas hydrates^20^ at 280 K for CO_2_ inclusion. Such moderate thermodynamic conditions are advantageous to develop gas separation process compared to chemical CO_2_ absorption by amine which usually requires high temperatures, e.g., 370–410 K to release the captured CO_2_^3,21^. Ionic clathrate hydrates form with water and ionic guest substances such as quaternary ammonium and phosphonium salts^22–27^. Among a vast variety of ionic guest substances, tetra-*n*-butylammonium bromide (TBAB), tetra-*n*-butylammonium chloride (TBAC), tetra-*n*-butylphosphonium bromide (TBPB) and tetra-*n*-butylphosphonium chloride (TBPC) are widely studied due to their less toxicity and good stability. The four butyl chains in their cations excellently fit into the cage-like network built by hydrogen-bonded water molecules. The anions also make bonds with the water molecules and compose a part of the network structure. These ionic clathrate hydrates have four basic structures, and they usually leave dodecahedral cages empty when they are formed under an atmospheric pressure^23^. The dodecahedral cages in the ionic clathrate hydrates are to incorporate small gas molecules such as CH_4_, N_2_, and CO_2_ under gas pressurized conditions.

Since CO_2_ is suitably incorporated in the ionic clathrate hydrates^28,29^, CO_2_ capture processes based on ionic clathrate hydrates were proposed^4,7,16,30–37^. CO_2_ capture from flue gas by ionic clathrate hydrates were reported a lot^4,6,32–37^. Particularly high CO_2_ selectivity was found in ionic clathrate hydrates^6,30,31^, although canonical gas hydrates also have the similarly sized-dodecahedral cages for gas occupancy. This is likely due to the distorted dodecahedral cages in the TBAB hydrates which incorporate CO_2_ more than the regular cages^28,29^. It was also suggested that CO_2_ storage capacity and selectivity of the TBAB hydrates irregularly depend on formation pressures because of the polymorphic phases of the TBAB hydrates, i.e., tetragonal and orthorhombic structures^6^. So far, while ionic clathrate hydrates were widely investigated, their gas separation properties and corresponding hydrate structures have not been studied. In comparison with other functional materials^38–40^, ionic clathrate hydrates have unique potential for gas capture and storage processes, because they are water-based and form rapidly under certain pressure and temperature conditions which can simplify the processes. To further design the ionic clathrate hydrates, their gas selectivity and capacity resulting from the hydrate structures need to be investigated regarding combinations of ionic guest substances and guest gas components.

In this work, we studied flue gas separation properties of ionic clathrate hydrates of TBAB, TBAC, TBPB, and TBPC which may form different structures. We performed gas separation tests by CO_2_ + N_2_ mixed gas of which compositions were ~0.15 and ~0.85 on a molar basis, respectively. The test pressures were 1, 3 and 5 MPa, and the subcooling temperatures which are driving force of hydrate formation were controlled to be within 2–4 K. Compositions of the aqueous solutions on a mass basis (*w*) were 0.200 for TBAB, TBAC, TBPB and TBPC, and 0.320 for TBAB. Single crystal X-ray diffraction and Raman spectroscopy were together used to characterize the hydrate structures. The gas separation tests found that these ionic clathrate hydrates have different CO_2_ selectivity and capacity: Large CO_2_ capacity for TBAB, TBPB and TBPC hydrate, and better CO_2_ selectivity for TBAC hydrates. The TBAB hydrates have irregularly high CO_2_ selectivity under 1 MPa of the formation pressure. Analyses by the X-ray diffraction and Raman spectroscopy clarified that such a variety of gas separation properties are caused by the structure of ionic clathrate hydrates. The present results suggest new potential for improving gas capacity and selectivity of ionic clathrate hydrates.

## Results

### Gas separation test

**Figure 1:**
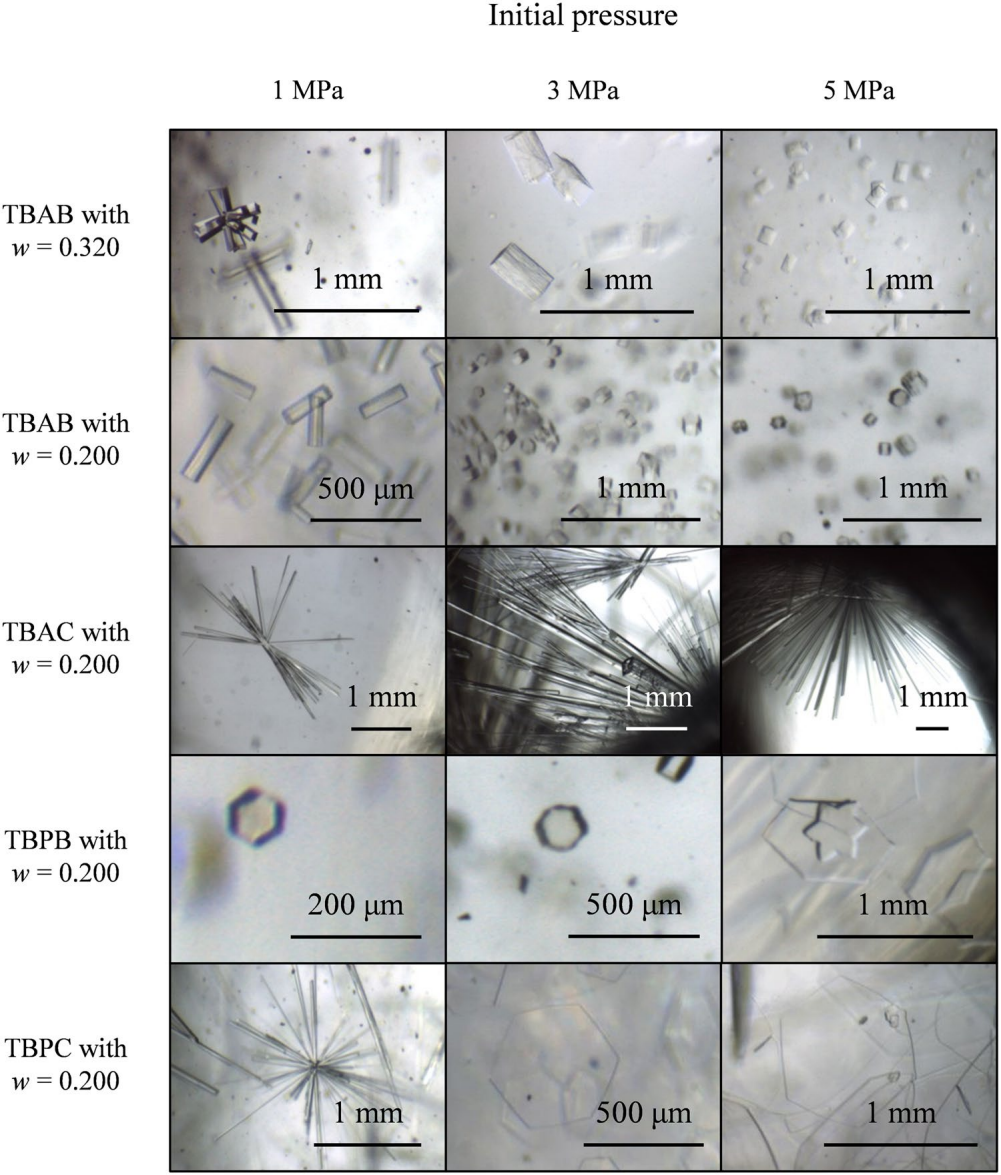
Single crystals of ionic clathrate hydrates formed in gas separation tests.

We performed gas separation tests twice in each system. During the tests, we optically observed morphologies of the hydrate crystals. Figure 1 shows pictures of the hydrate crystals at the beginning of the crystallization. The TBAB hydrates formed with *w* = 0.200 had a columnar shape. These crystals formed at 3 and 5 MPa were smaller than that formed at 1 MPa. This is due to increase of the formation rate at higher pressures as shown in Supplementary Figures S3–S5. These TBAB hydrate crystals had square or hexagonal sections, which imply that they have the polymorphism even under the gas pressures as well as at atmospheric pressure^41–46^. Morphologies of the TBAB hydrates formed with *w* = 0.320 basically followed with those with *w* = 0.200, however, clusters of the columnar crystals were observed at 1 MPa which are similar to the TBAC hydrate crystals. The TBAC hydrates formed with *w* = 0.200 were thin columnar-shaped crystals which radially grew and clustered. They had square sections as well as the TBAB hydrates. The thickness of the TBAC hydrate crystals was not obviously changed due to the formation pressures. The TBPB hydrate crystals formed with *w* = 0.200 had hexagonal sections and they became wider and thinner as the initial pressure increased. At 5 MPa, the hexagram shaped crystal was found as shown in Figure 1. This particular shape was likely due to a high crystal growth rate at the beginning of crystallization. The TBPC hydrate formed with *w* = 0.200 basically had the same tendency as the TBPB hydrates, however, the TBPC hydrate crystals made clusters of columnar crystals under 1 MPa as well as the TBAC hydrate crystals. Such complicated morphological behavior may be related to two-stage gas uptake shown in Supplementary Figures S3–S5 in Supplementary Information. At the test with 1 MPa of the initial pressure, the TBPC hydrates once captured similar amount of gas as the TBAC hydrates, but the TBPC hydrates further captured the gas as much as the TBAB and TBPB hydrates. While in the case of TBPC hydrates such two-stage gas uptake disappeared at higher pressure levels, the two-stage gas uptake was observed for TBAB hydrates with *w* = 0.320 at all the three initial pressures.

**Figure 2:**
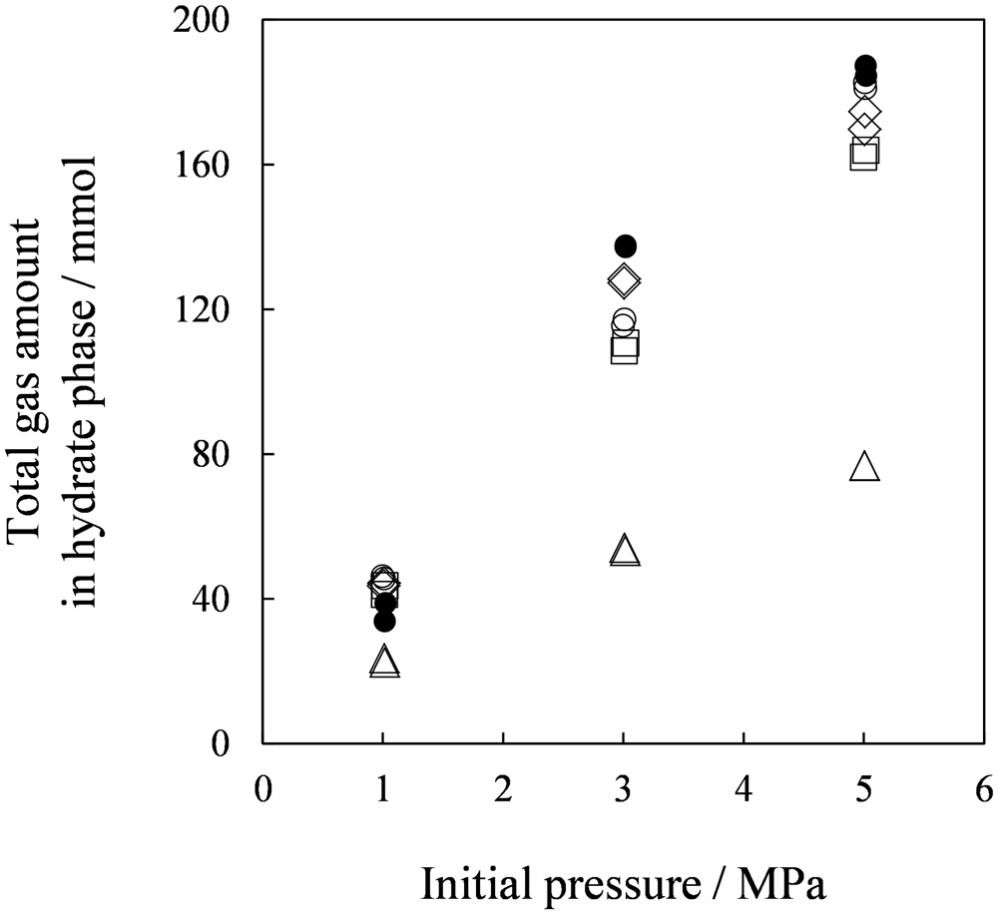
Gas amount captured by ionic clathrate hydrates. The symbols show ionic guest substances: ●, TBAB with *w* = 0.320; ○, TBAB with *w* = 0.200; △, TBAC with *w* = 0.200; □, TBPB with *w* = 0.200; ◊, TBPC with *w* = 0.200.

**Figure 3:**
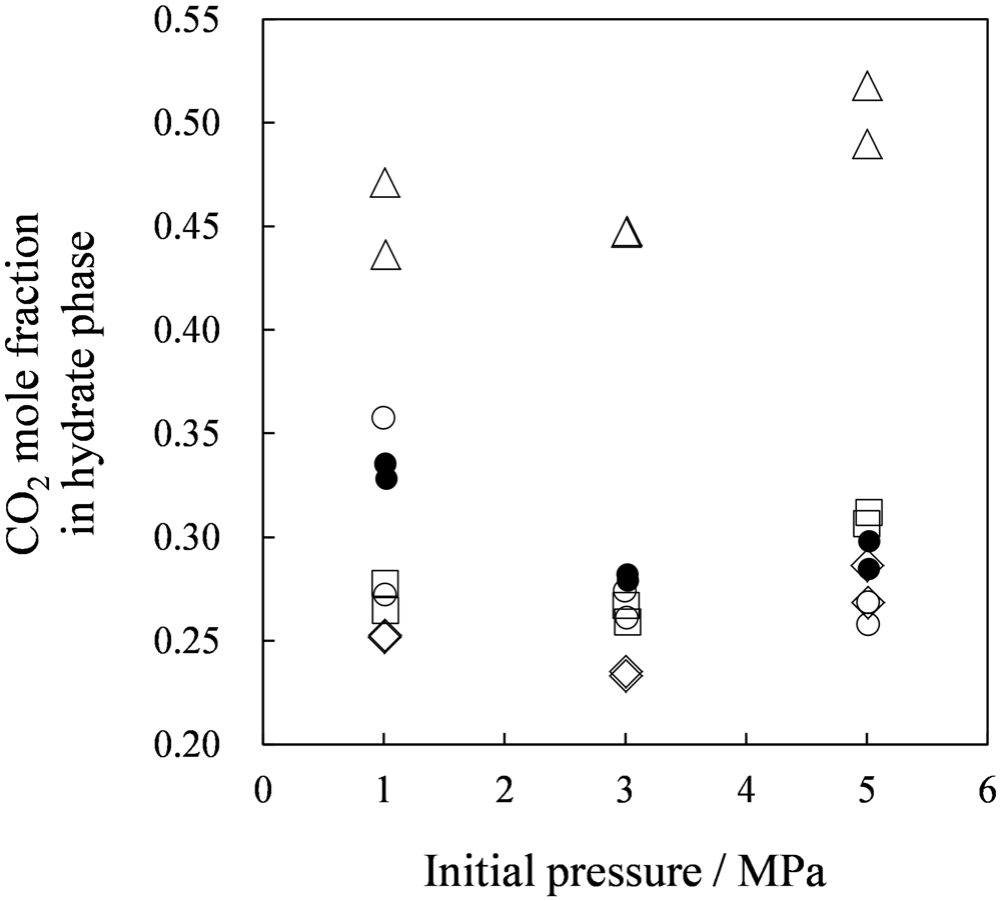
CO_2_ mole fraction in hydrate phase. The symbols show ionic guest substances: ●, TBAB with *w* = 0.320; ○, TBAB with *w* = 0.200; △, TBAC with *w* = 0.200; □, TBPB with *w* = 0.200; ◊, TBPC with *w* = 0.200.

**Figure 4:**
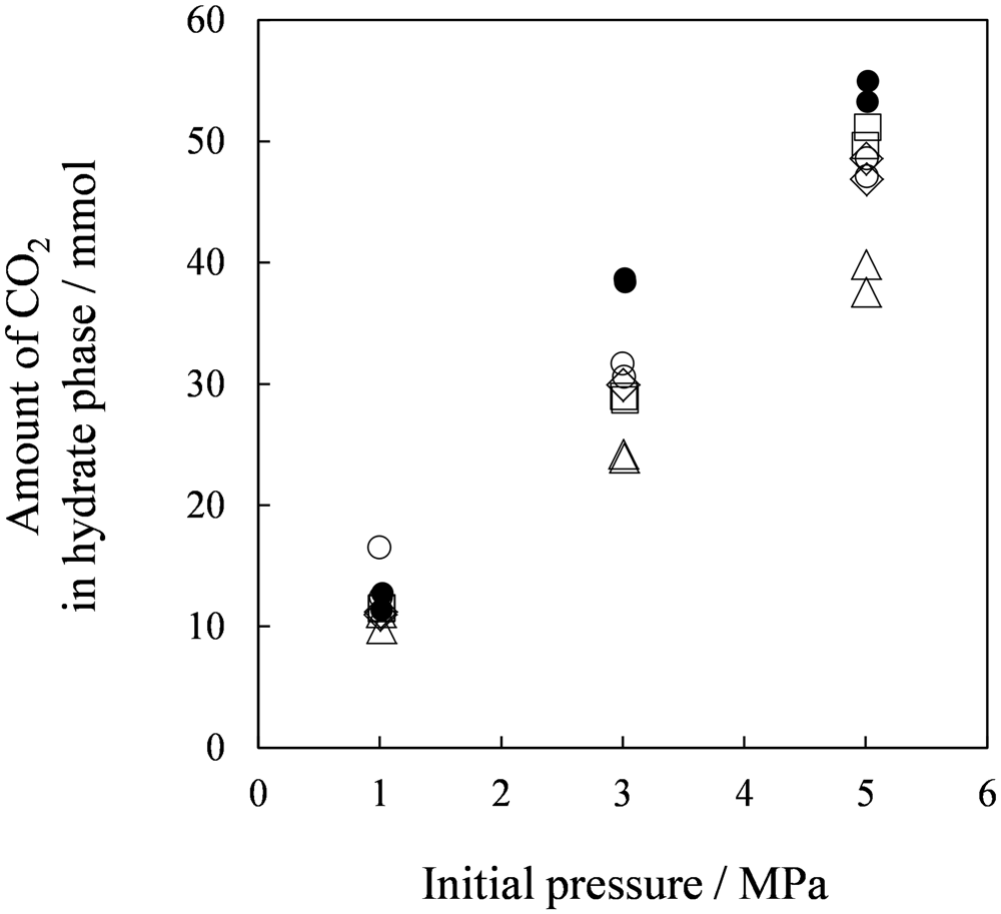
CO_2_ amount captured by ionic clathrate hydrates. The symbols show ionic guest substances: ●, TBAB with *w* = 0.320; ○, TBAB with *w* = 0.200; △, TBAC with *w* = 0.200; □, TBPB with *w* = 0.200; ◊, TBPC with *w* = 0.200.

Figure 2 shows total gas (CO_2_ + N_2_) amounts in the hydrate phases. The amounts captured in the TBAB hydrate formed with *w* = 0.320 were the largest among the tested materials. While the TBPB hydrates and the TBPC hydrates captured the gas as much as the TBAB hydrates, the TBAC hydrates captured the least which was less than a half amount of the gas the other hydrates captured. Figure 3 shows CO_2_ mole fraction in the hydrate phases. The CO_2_ mole fraction in the TBAC hydrates were about double of those in the other hydrates, while the total gas amounts captured in these hydrates were a half approximately. These results indicated that the CO_2_ selectivity against N_2_ for the TBAC hydrates was better than the TBAB, TBPB and TBPC hydrates. Although comparable CO_2_ mole fractions in the TBAB, TBPB and TBPC hydrates were obtained, the TBAB hydrates had irregularly better CO_2_ selectivity at 1 MPa. This result showed an additional advantage of TBAB hydrates for the gas separation process operated at low pressures^6,7^. The TBPB and TBPC hydrates showed slightly lower CO_2_ mole fractions in the hydrate phase. Based on the results for amounts and compositions of the captured gas, we determined the amount of CO_2_ in the hydrate phases as shown in Figure 4. Consequently, the data for the TBAC hydrates and the other hydrates were almost equalized in contrast to the distinct total gas amount (Figure 2). This Figure shows that the CO_2_ amount captured by the hydrates linearly increased depending on the initial pressure. At each pressure level, the TBAB hydrates captured the largest amount of CO_2_. While the CO_2_ mole fractions in the TBAC hydrate phase were the highest, the CO_2_ amounts captured by the TBAC hydrates were as much as 60–90% of those captured by the TBAB, TBPB and TBPC hydrates. Kim and Seo^37^ reported that the gas storage capacity of TBAC hydrates was larger than that in TBAB hydrates. Because they used aqueous solutions of which compositions correspond to stoichiometric compositions of their tetragonal structure hydrates^24,43,47,48^ differing from our tests, the tetragonal structure hydrates may dominantly form in the both systems. This comparison suggests that gas capacity of ionic clathrate hydrates varies by ionic guest substances and their compositions in aqueous phase.

The compositions of the aqueous solutions were measured before and after the gas separation tests. The method and results were detailed in Supplementary Table S1 in Supplementary Information. At the tests with TBAB with *w* = 0.320, the TBAB compositions in the aqueous phase changed little. This is likely due to that the orthorhombic TBAB hydrate congruently formed at *w* = 0.32 based on the stoichiometric compositions of the TBAB hydrates for the orthorhombic structure (*w* = 0.32) and the tetragonal structure (*w* = 0.40). The other results for TBAB, TBAC, TBPB and TBPC hydrates with *w* = 0.200 showed lowering of the composition due to their stoichiometric compositions of the hydrate crystals^24,26,42,47^. These data were nearly independent from the initial pressures, which suggests that almost the same amount of the hydrates formed with the three initial pressures. Taking account of the fact that the captured gas amounts increased as the initial pressure raised (see Figure 2), it was found that more gas was incorporated in the unit volume of the hydrates at higher pressures.

### Single crystal X-ray diffraction

**Figure 5:**
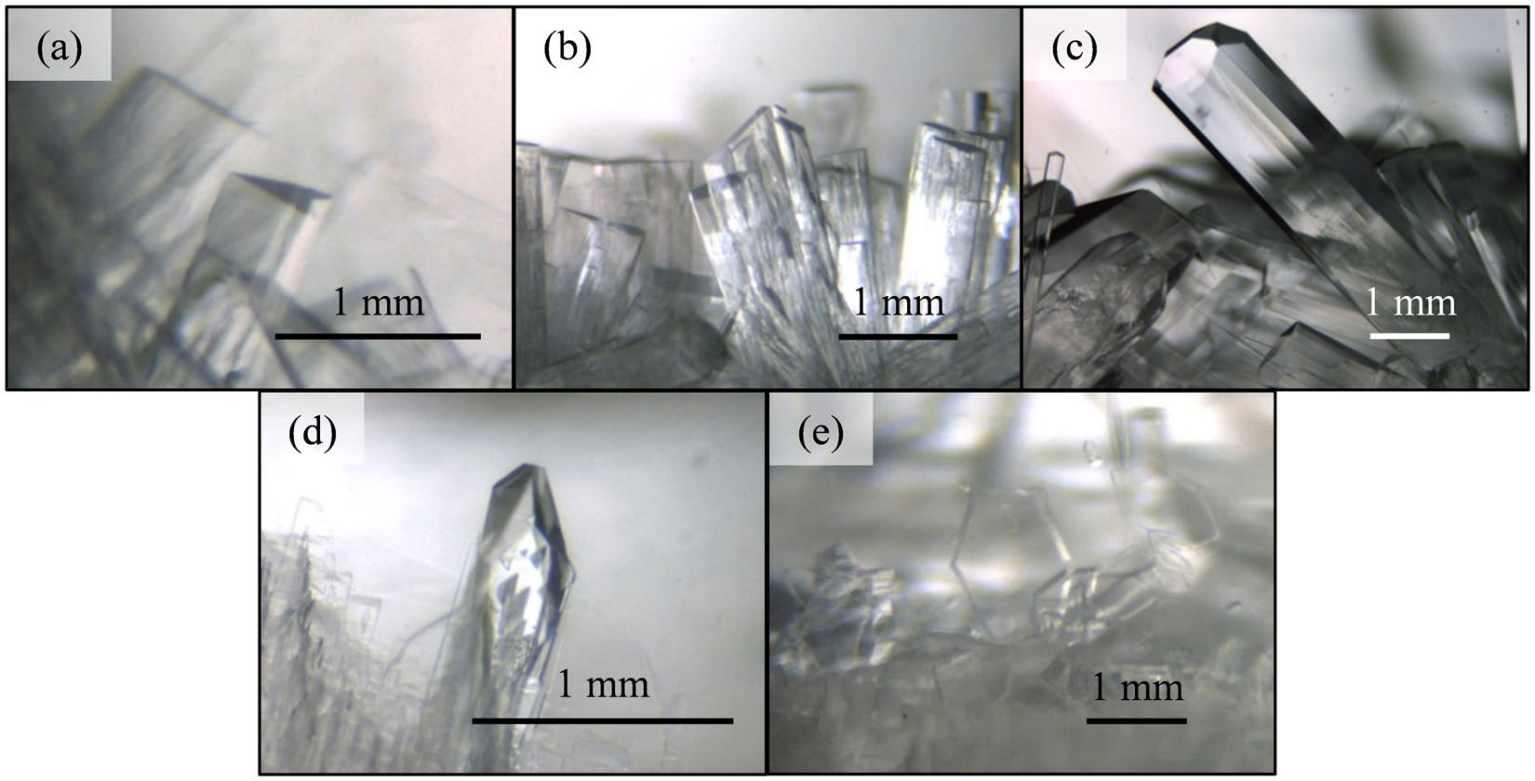
Single crystals of ionic clathrate hydrates formed with CO_2_ + N_2_ under static conditions. (**a**) CO_2_ + N_2_ + TBAB hydrate with *w* = 0.320 formed at 286.6 K and 5.08 MPa; (**b**) CO_2_ + N_2_ + TBAB hydrate with *w* = 0.200 formed at 284.2 K and 5.04 MPa; (**c**) CO_2_ + N_2_ + TBAC hydrate with *w* = 0.200 formed at 287.2 K and 5.03 MPa; (**d**) CO_2_ + N_2_ + TBPB hydrate with *w* = 0.200 formed at 285.2 K and 4.93 MPa; (**e**) CO_2_ + N_2_ + TBPC hydrate with *w* = 0.200 formed at 285.2 K and 5.09 MPa.

To identify the hydrate structure in each system by X-ray diffraction and Raman analyses, we formed single crystals as shown in Figure 5. With the stationary conditions without mixing the phases, the columnar shaped thick crystals mostly formed as reported in the literature^18,19,26,49^. We performed X-ray diffraction for the single crystals of TBAB, TBAC, TBPB and TBAC hydrates formed with *w* = 0.200. The crystal data are summarized in Table 1 and detailed in Supplementary Table S3 in Supplementary Information. For the TBAB hydrates the orthorhombic structure with a space group of *Imma* was found, which agreed with our previous studies for TBAB hydrates containing CO_2_ gas^28^. This result suggests that ~10% of CO_2_ in the mixed gas is sufficient to induce the symmetry-lowered structure of the orthorhombic TBAB hydrate^28^. The orthorhombic structure was also found for the TBPC hydrates, but with an irregular space group: *Cmmm*. Limited quality of the present data, it was not clear that the combination of these substances induces this base-centered lattice. The tetragonal hydrate structure was found for the TBAC hydrate. This is consistent with the crystal structure of TBAC hydrate formed under atmospheric pressure^47^. The crystal structure of the TBPB hydrate was not clearly determined in this study because of its less crystallinity: The presently found lattice was the hexagonal, but is likely the orthorhombic lattice which is originally transformed from the hexagonal lattice^22,23^. Based on the stoichiometry of the vacant cages available for gas occupancy^22,23^, the gas capacity of the tetragonal hydrate is smaller than that of the orthorhombic hydrate^28,50^. The present results showed that the TBAC hydrates only formed the tetragonal structure differing from the other TBAB, TBPB and TBPC hydrates. This fact also supports that the amounts of the gas captured by the TBAC hydrates were distinctly small (see Figure 2), while the amounts of the formed hydrates were similar according to the aqueous solution analyses. Also, such a structural difference between the orthorhombic and the tetragonal hydrates may be a plausible reason for the superior CO_2_ selectivity of the TBAC hydrates.

**Table 1:** Crystallographic data and structural refinement parameters for the single crystals of ionic clathrate hydrate formed in this study.

	CO_2_ + N_2_ + TBAB hydrate with *w* = 0.200	CO_2_ + N_2_ + TBAC hydrate with *w* = 0.200	CO_2_ + N_2_ + TBPB hydrate with *w* = 0.200	CO_2_ + N_2_ + TBPC hydrate with *w* = 0.200
Lattice	orthorhombic	tetragonal	hexagonal (Possibly orthorhombic)	orthorhombic
Space group	*Imma*	*P*4_2_/*m*	(Not determined)	*Cmmm*
*a* (Å)	21.419(4)	23.870(3)	12.0602(17)	12.036(2)
*b* (Å)	25.833(5)	23.870(3)	12.0602(17)	21.145(4)
*c* (Å)	12.218(2)	12.497(3)	12.585(3)	12.685(3)
*R*_*int*_/*Rσ*	0.1211/0.0765	0.0758/0.0446	0.1302/0.0427	0.1087/0.0592

### Raman spectroscopy

**Figure 6:**
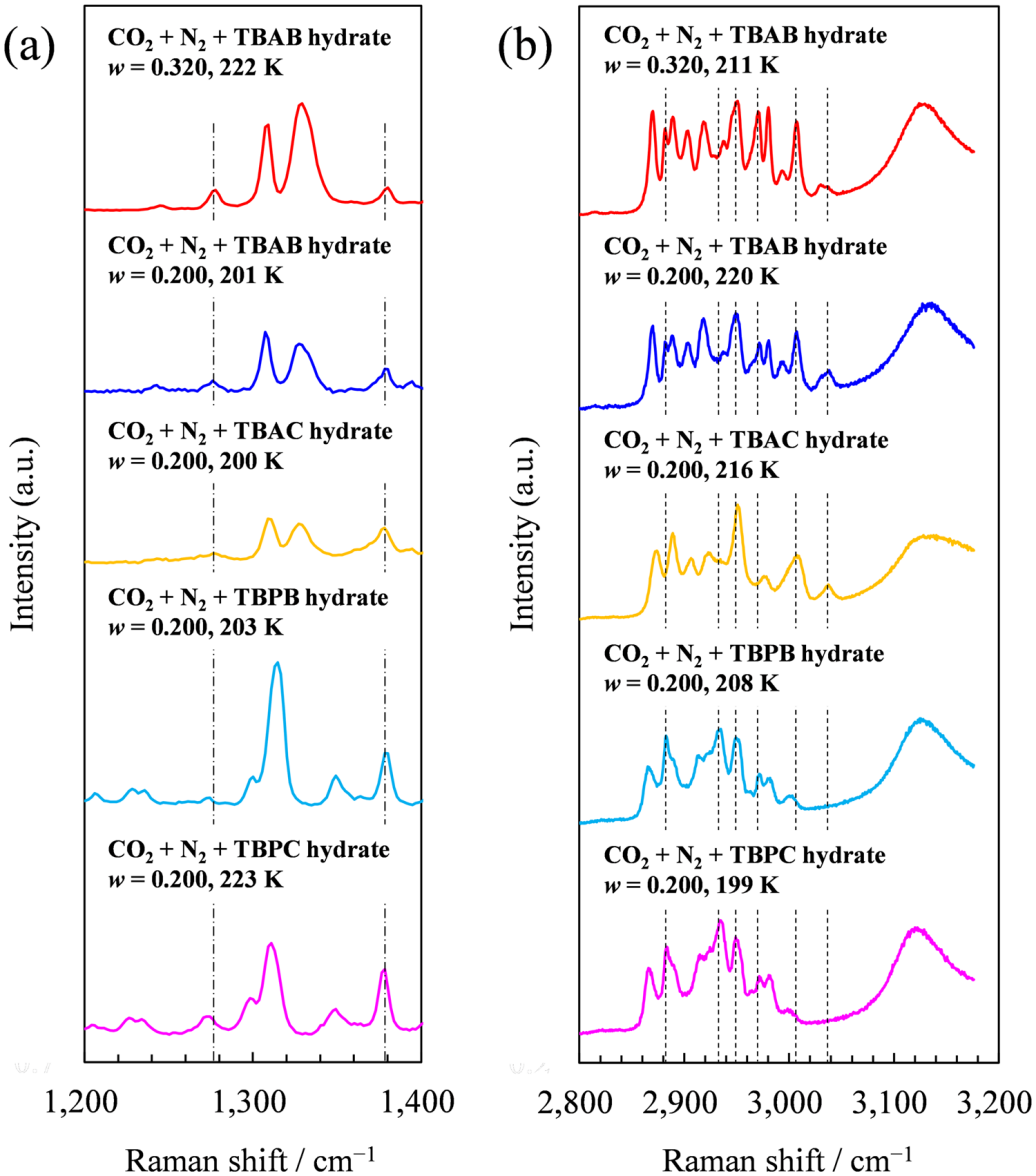
Raman spectra of the ionic clathrate hydrates formed with CO_2_ + N_2_ gas. Spectra were collected under N_2_ atmosphere at 199–223 K with resolutions of 1.8 cm^−1^ for (**a**) and 0.6 cm^−1^ for (**b**). Chain lines indicate CO_2_ peaks at 1278 and 1380 cm^−1^. Dashed lines indicate the peaks of C–H stretching vibration modes of TBA^+^ and/or TBP^+^ at 2884, 2937, 2947, 2969, 3009 and 3040 cm^−1^.

We have conducted Raman spectroscopy measurements on the single crystals of the present ionic clathrate hydrates. To confirm the presence of the gas in the hydrate phase, the hydrates formed under atmospheric pressure were together tested. The pictures of these crystals were provided in Supplementary Figure S6. The collected Raman spectra are fully provided in Supplementary Figure S7. Figure 6 summarizes obtained vibrational Raman spectra for the hydrates formed with CO_2_ + N_2_ gas. When CO_2_ is trapped in the hydrate cages, the two peak positions at around 1273 cm^−1^ and 1380 cm^−1^ slightly shift to lower frequency from those in gas phase regardless of their hydrate structures^43,51–56^. In Figure 6, the two CO_2_ peaks appeared in the five hydrate samples formed with CO_2_ + N_2_ gas. The lower peaks were at 1273 cm^−1^ for TBPC hydrate with *w* = 0.200; at 1274 cm^−1^ for TBPB hydrate with *w* = 0.200; at 1276 cm^−1^ for TBAB and TBAC hydrate with *w* = 0.200; at 1278 cm^−1^ for TBAB hydrate with *w* = 0.320. The higher peaks were at 1378 cm^−1^ for TBAB, TBAC, TBPB or TBPC hydrate with *w* = 0.200; at 1380 cm^−1^ for TBAB hydrate with *w* = 0.320. They were not observed in the hydrates formed under atmospheric pressure, which clearly shows sufficient amounts of CO_2_ were captured in the hydrates under the pressurized conditions. Although the higher CO_2_ peaks were clearly observed, the lower peaks were not clear. By collecting Raman spectra of CO_2_ + N_2_ + tetrahydrofuran (THF) hydrate which is a canonical structure II gas hydrate, we confirmed that the present CO_2_ peaks observed with the ionic clathrate hydrates were occurred by the CO_2_ incorporation in the hydrate phases. As shown in Figure S7, the CO_2_ peaks appeared at 1276 and 1382 cm^−1^ which agreed with the literature data^17,54,56^. The CO_2_ peaks in the THF hydrate are basically consistent with those in the present ionic clathrate hydrates except for slight shift in the lower CO_2_ peak in the TBPB and TBPC hydrates. A lot of works by Raman spectroscopy were performed to identify the biphase of the TBAB hydrate^13,43,45,46^. A range between 2850–3050 cm^−1^ includes C–H stretching vibration modes of TBA^+^ or TBP^+ ^^43,45,46,57–59^ where the structural difference between the orthorhombic and tetragonal TBAB hydrate structures was found^13,43,45,46^. The TBAC hydrate and the TBPB hydrate were reported to have the tetragonal hydrate structure and the orthorhombic hydrate structure, respectively^26,47^, without polymorphism. Therefore, they are also useful to identify the orthorhombic and tetragonal structures. The presently collected spectra between 2850–3050 cm^−1^ for the TBAC and TBPB hydrates showed similar peak patterns in Figure 6b. The spectra for the TBPC hydrate in Figure 6b were similar to those for the TBPB hydrate. Consequently, these hydrates may have the orthorhombic structure. The spectra for the TBAB hydrate in Figure 6b were not identical with those for the others, but contained unique peaks of both of the tetragonal TBAC hydrate and the orthorhombic TBPB and TBPC hydrate, e.g., peaks at 2884, 2937, 2947, 2969, 3009 and 3040 cm^−1^. This fact also suggests the biphasic TBAB hydrate phase in the present samples.

## Discussion

Our suites of analyses gave some new information for developing gas separation process based on ionic clathrate hydrates. As shown by our X-ray diffraction and Raman analyses, the TBAB hydrates may have the mixed phase containing both the orthorhombic and tetragonal structure hydrates when they were formed under the CO_2_ + N_2_ gas pressures. This is likely reason for their irregularly high CO_2_ selectivity at 1 MPa (see Figure 3), because the tetragonal structure of the TBAB hydrates are more stable at the relatively low test temperature at 1 MPa compared to those at the other two test pressures. For the TBAC, TBPB and TBPC hydrates, the data obtained by both X-ray diffraction and Raman analyses showed that each hydrate consistently had the uniform structure: Tetragonal structure for the TBAC hydrates and orthorhombic structure for the TBPB and TBPC hydrates. This interpretation also agrees with the gas capacity data obtained at the gas separation tests (see Figures 2 and 4) which had linearly increasing tendencies depending on the test pressure and no irregularity as found for the TBAB hydrates.

Compared to our previous work^6^ in the small scale (~200 cm^3^ reactor), the separation factors of the TBA and TBP salt hydrates obtained in this work were lower, although the tendency for TBAB hydrates was similar. This suggests that CO_2_ selectivity of these hydrates degrades as the driving force for hydrate formation decreases due to the pressure drop in the batch test system. On the contrary, significant improvement by means of small reaction field process with porous materials and continuous gas feed process which can maintain the driving force are expected^60,61^.

In conclusion, we tested four different ionic clathrate hydrates, i.e., TBAB, TBAC, TBPB and TBPC hydrates, for CO_2_/N_2_ gas separation, and the results clearly showed that these materials have unique gas capacity and CO_2_ selectivity. Our X-ray diffraction and Raman analyses characterized the hydrate structures. The TBAC hydrates had the tetragonal hydrate structure which may bring remarkably high CO_2_ selectivity. The TBAB hydrates likely had the biphase, i.e., tetragonal and orthorhombic structures, especially at 1 MPa of the initial pressure. This mixed phase captured CO_2_ more than the TBPB and TBPC hydrates which may form only the orthorhombic structure. Although the orthorhombic structure hydrate has the largest gas capacity among the basic four structures of ionic clathrate hydrates^22,23,50^, this study showed that the tetragonal hydrate captured CO_2_ more efficiently than the orthorhombic hydrate. These facts suggest new potential for improving gas capacity and selectivity of ionic clathrate hydrates by choosing suitable ionic guest substances for guest gas components. Further analyses on such as gas occupancy in hydrate phases and hydrate formation kinetics are necessary to fully understand the presently found structure-driven gas separation properties and to design ionic clathrate hydrates.

## Methods

### Materials

We used two CO_2_ + N_2_ mixed gases of which compositions were 0.1524 and 0.8476, 0.1502 and 0.8498 in mole fraction, respectively (Takachiho Chemical Industrial Co., LTD., Tokyo). Ionic guest substances we used in this study were TBAB with certified purity of ≥0.99 on mass basis (Sigma-Aldrich, Co., Missouri), TBAC with certified purity of ≥0.97 in mass fraction (Sigma-Aldrich, Co., Missouri), TBPB with certified purity of ≥0.98 in mass fraction (Sigma-Aldrich, Co., Missouri) and TBPC with certified purity of ≥0.96 in mass fraction (Sigma-Aldrich, Co., Missouri). THF used in this study had certified purity of 0.999 in mass fraction (Sigma-Aldrich, Co., Missouri). We used water which was deionized, filtrated by activated carbon and sterilized by an ultra-violet lamp. The resistivity and total organic content of the used water were ≥18.2 MΩ and ≤5 ppb, respectively. Aqueous solutions were gravimetrically prepared using an electronic balance (GX-6100, A&D Co., Tokyo) with 0.30 g of uncertainty with 95% reliability.

### Gas separation tests

An apparatus we used for gas separation test provided in Supplementary Figure S1 in Supplementary Information mainly consisted of a hydrate formation reactor, a water bath made of polymethyl methacrylate (PMMA), a proportional-integral-derivative (PID) controlled heater and a cooler. The hydrate formation reactor had 800 cm^3^ of an inner volume. The reactor was equipped with two glass windows for observing its inside, a strain-gauge pressure sensor (VPRTF-A2-10MPaW-5, Valcom, Co. LTD., Osaka, Japan), a platinum resistance thermometer inserted from the bottom (Pt 100 Class B 2 mA, NRHS1-0, Chino, Co., Tokyo), a sealed tube, and an electromagnetically induced stirrer on the lid. Gas compositions were analyzed by a gas chromatograph (GC-2014, Shimadzu, Co., Kyoto, Japan). We used a refractometer (PR-RI, ATAGO Co., LTD., Tokyo) for composition analysis for aqueous solution. The measurement accuracy of the refractometer for temperature and refractive index was 1 K and 0.0002, respectively. The uncertainty for this measurement was estimated to be 0.001 in mass fraction.

We employed three different initial hydrate formation pressures: 1, 3 and 5 MPa. We maintained system temperatures to control the subcooling temperatures to be 2–4 K at corresponding initial pressures. The subcooling temperatures were determined based on the phase equilibrium data provided in Supplementary Information and on our previous study^6^. Compositions of aqueous solutions were *w* = 0.200 for TBAB, TBAC, TBPB and TBPC, and *w* = 0.320 for TBAB which corresponds to the stoichiometric composition of the orthorhombic TBAB hydrates. We supplied 300 g of the aqueous solution into the reactor. To eliminate residual air in the reactor, the reactor was firstly evacuated by a vacuum pump and charged-discharged with the CO_2_ + N_2_ mixed gas with 1 MPa for three times. We repeated this process for three times. After dissolution of the gas was completed with the aid of stirring, we sampled the CO_2_ + N_2_ mixed gas in a 10 cm^3^-cylinder with ~0.2 MPa at a room temperature. We stopped the stirrer and cooled down the sealed tube up to 260–270 K to promote hydrate nucleation by inserting a metal rod quenched by liquid nitrogen. Hydrate formation lasted for 20 hours approximately on all the tests. The gas inside the reactor was sampled again after the hydrate formation. The compositions of gas were analyzed by a gas chromatograph (GC-2014, Shimadzu, Co., Kyoto, Japan) equipped with a thermal conductivity detector. Argon gas (≥0.99999 in mole fraction certified purity, Taiyo Nippon Sanso, Co., Tokyo) and the separation column (Shincarbon ST 50/80, Shimadzu, Co., Kyoto, Japan) were used for the analyses. The details of calculation process of gas compositions in the hydrate phase were described in our previous paper^6^. Compositions of the aqueous solution were measured after the hydrate formation by the refractometer. After the test, for hydrate dissociation, the system temperature was maintained to be at a temperature which is 5 K higher than the equilibrium temperature for at least ~2 hours. Subsequently, the system temperature was decreased to be a test temperature for the second test. The measurement uncertainties for temperature, pressure, *w* and gas phase composition in gas separation tests are 0.3 K, 0.03 MPa, 0.001 in mass fraction and 0.006 in mole fraction, respectively with 95% reliability.

### Single crystal X-ray diffraction

For single crystal hydrate formation under a gas pressure, we used an apparatus consists of a hydrate formation reactor, a temperature controlled bath, a pressure sensor (GP-M100, KEYENCE, Co., Osaka, Japan) and a thermometer (EcoScan Temp 6, Eutech Instruments Pte Ltd., Singapore). About 3 g of an aqueous solution of ionic guest was supplied into the reactor. After three times repetition of evacuating the reactor and charging/recharging with the CO_2_ + N_2_ mixed gas with 1 MPa, CO_2_ + N_2_ mixed gas was injected into the reactor. The initial formation pressure was ~5 MPa, and the temperature was maintained to control the subcooling temperature to be 2–4 K at the initial pressure. After the sufficient crystals grew, they were separated from the liquid, and cooled to ~250 K. The pressure of the reactor was released, and the crystals were taken out from the reactor. For single crystal hydrate formation under atmospheric pressure, we followed the similar manner and used a similar apparatus but with a glass tube instead of the high pressure reactor. The measurement uncertainties with 95% reliability for temperature and pressure were 0.3 K and 0.02 MPa, respectively.

A single crystal was selected and sized under cold nitrogen atmosphere at below 250 K, and subjected to X-ray diffraction measurements. We used an imaging plate-type X-ray diffractometer (R-AXIS -RAPID-S, Rigaku, Co., Tokyo) with a Mo K*α* radiation source (wave length: 0.71073 Å). The measurement temperature was 123(1) K. Measurement parameters are provided in Supplementary Information in detail.

### Raman spectroscopy

We used Raman spectrometer (inVia Raman Microscope, Renishaw plc., Gloucestershire) equipped with 3000 and 1800 grooves/mm gratings with resolutions of 0.6 and 1.8 cm^−1^, respectively, a laser source having a 514.5 nm (Stellar-pro, Modu-Laser, LLC., Utah), and an objective lens of nine magnifications (Atago Bussan Co., LTD., Tokyo). Raman spectra of the samples were collected in an insulated box made of foaming polystyrene at 193–223 K under cold N_2_ vapor from liquid nitrogen. A single crystal of silicon was used for wave number calibration.

## Electronic supplementary material

Supplementary Information
