# Supplementary material for: Structure-driven CO2 selectivity and gas capacity of ionic clathrate hydrates
Source: Sci Rep. 2017 Dec 8;7:17216. doi: 10.1038/s41598-017-17375-1 (PMC5722917; doi:10.1038/s41598-017-17375-1)
Supplement: Supplementary file 1 — Supplementary Information [file 41598_2017_17375_MOESM1_ESM.pdf]

Supplementary information for:

Structure-driven CO_2 selectivity and gas capacity of ionic clathrate hydrates

Hidenori Hashimoto[1,2], Tsutomu Yamaguchi[1,2], Hiroyuki Ozeki[1], Sanehiro Muromachi[2]*

[1]Graduate School of Environmental Science, Toho University, 2-2-1 Miyama, Funabashi-shi, Chiba 274-8510, Japan

[2]Research Institute for Energy Frontier (RIEF), National Institute of Advanced Industrial Science and Technology (AIST), 16-1 Onogawa, Tsukuba 305-8569, Japan

* Author to whom correspondence should be addressed. (S. M.)

E-mail: s-muromachi@aist.go.jp

Tell: +81-29-861-4287

Fax: +81-29-861-8706

1. Apparatus of gas separation test

Figure S1 shows a schematic of apparatus used for the gas separation test.

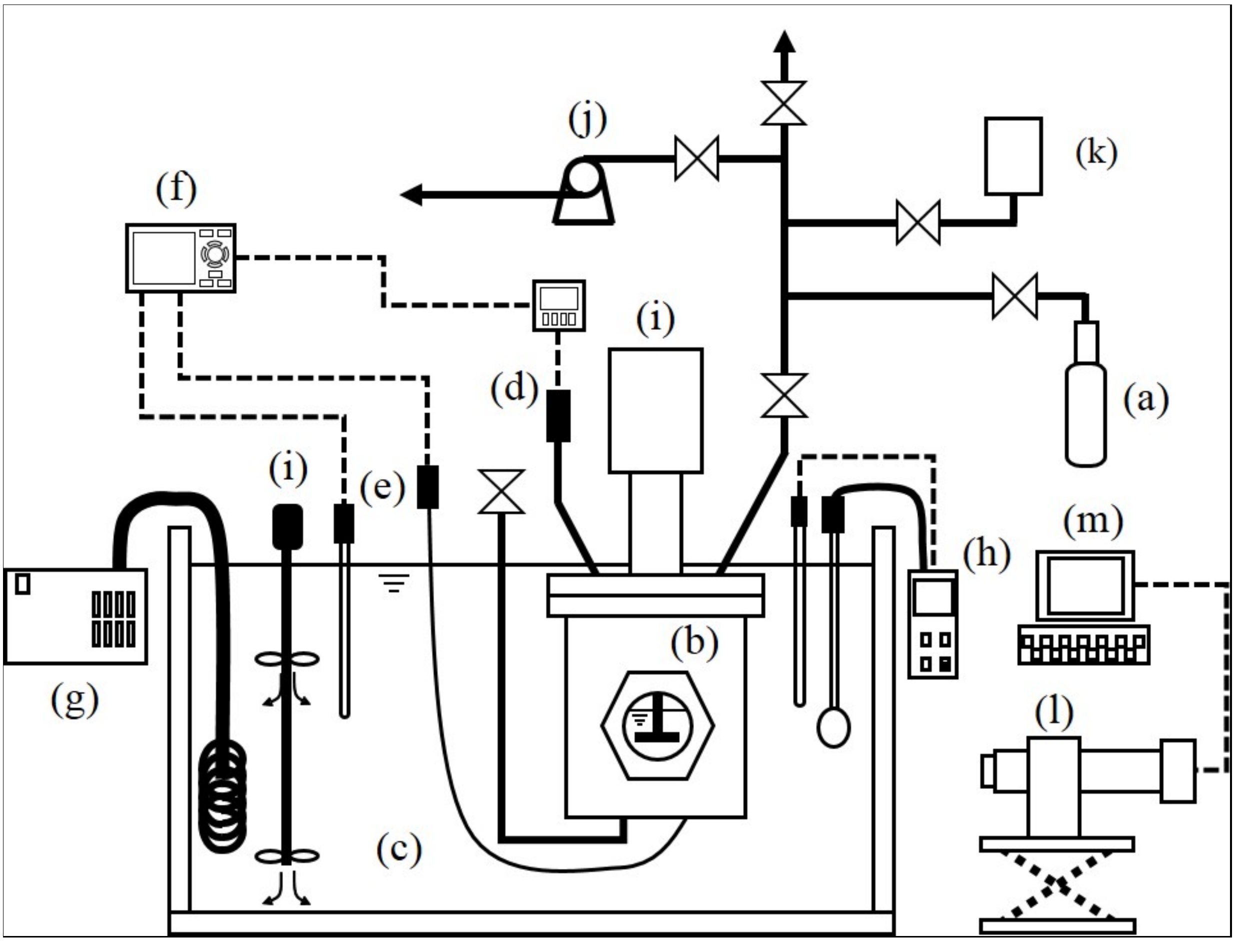

Figure S1. Schematic of apparatus used for gas separation test. (a) Gas cylinder, (b) Hydrate formation reactor, (c) PMMA water bath, (d) Pressure sensor, (e) Thermometer, (f) Data logger, (g) Cooler, (h) PID controlled heater, (i) Magnetic stirrer, (j) Vacuum pump, (k) Gas sampling vessel, (l) Microscope; (m) PC.

2. Phase equilibrium measurements for CO_2 + N_2 + (TBAB, TBAC, TBPB or TBPC) hydrate

To determine subcooling temperatures of gas separation tests which are driving force of hydrate formation, we performed phase equilibrium measurements for CO_2 + N_2 + (TBAB, TBAC, TBPB or TBPC) + H_2O system at around 1, 3 and 5 MPa. We employed isochoric method[S1, S2] with 0.5 K of a temperature increment. CO_2 + N_2 mixed gas was injected into the reactor. Mixing by the magnetic stirrer was started for gas dissolution into the aqueous phase. After the measurements, we analyzed the gas composition by the gas chromatograph (GC-2014, Shimadzu, Co., Kyoto, Japan). The obtained CO_2 compositions were consistent within 0.134–0.138 in mole fraction. Results of the phase equilibrium measurements are summarized in Figure S2.

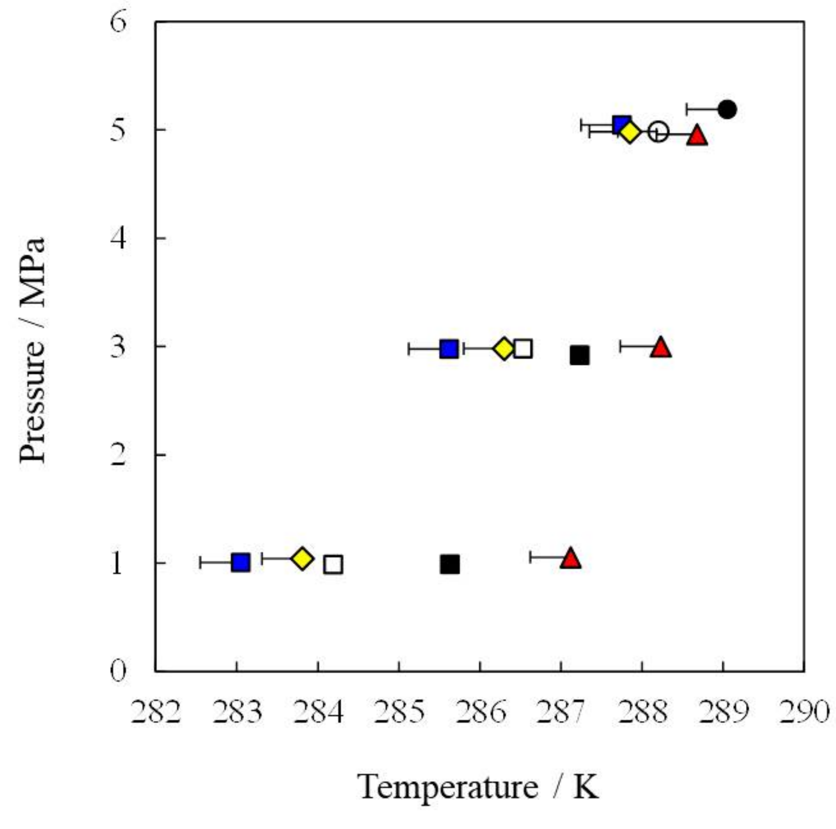

Figure S2. Three phase equilibrium data for CO_2 + N_2 + (TBAB, TBAC, TBPB or TBPC) hydrates. The symbols indicate aqueous solutions: ●, TBAB with *w* = 0.320; ○, TBAB with *w* = 0.200; △ with red, TBAC with *w* = 0.200; □ with blue, TBPB with *w* = 0.200; ◊ with yellow, TBPC with *w* = 0.200; ■, TBAB with *w* = 0.320 [S3]; □, TBAB with *w* = 0.200 [S3].

3. Detailed results of gas separation tests

We performed gas separation tests twice in each system. Two graphs in Figure S3–S5 show the pressure trends of the first tests and the subsequent second tests at each pressure level. During the gas separation tests by TBAB with w = 0.320, the pressure decreases on two stages. The irregular pressure decrease was also observed during the test of TBPC with w = 0.200 at 1 MPa.

For the aqueous solution measurement before and after the gas separation tests, we correlated refractive index to w. We used aqueous solutions of the ionic guest substances having twelve different w between 0.050 and 0.600. Table S1 summarises the results. For the gas separation test with the TBAB hydrates at 1 MPa, it was not possible to sample aqueous solution because of a tube plugging by the hydrates.

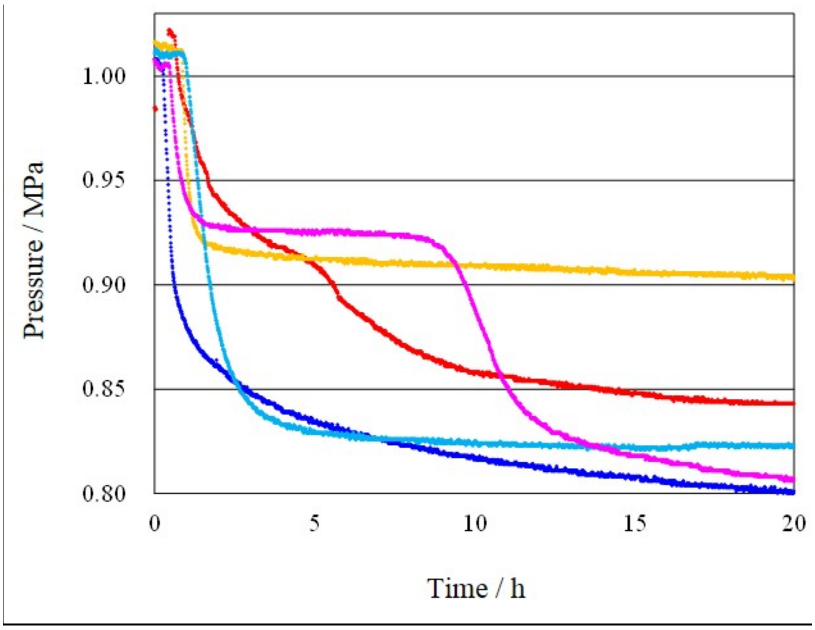

(a) First test

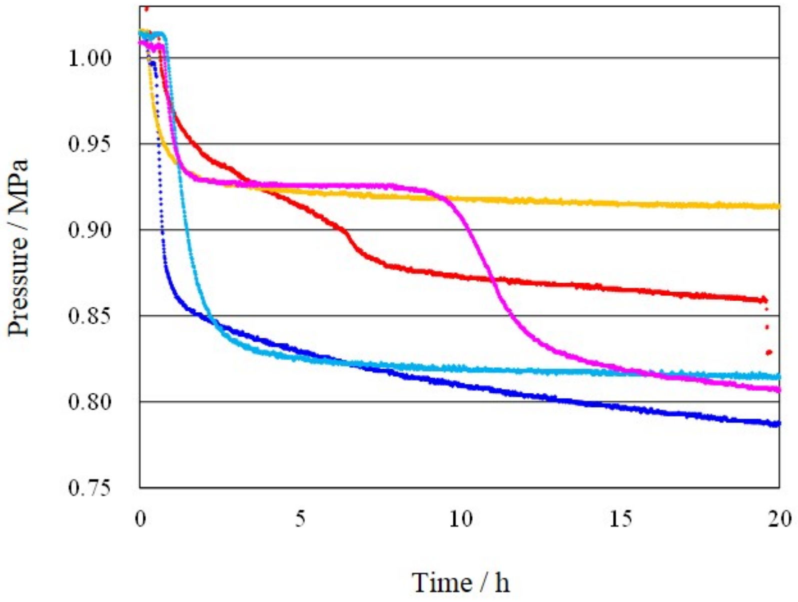

(b) Second test

Figure S3. Pressure trends of gas separation tests at 1 MPa. Colours: red, TBAB with *w* = 0.320; blue, TBAB with *w* = 0.200; orange, TBAC with *w* = 0.200; light blue, TBPB with *w* = 0.200; pink, TBPC with *w* = 0.200

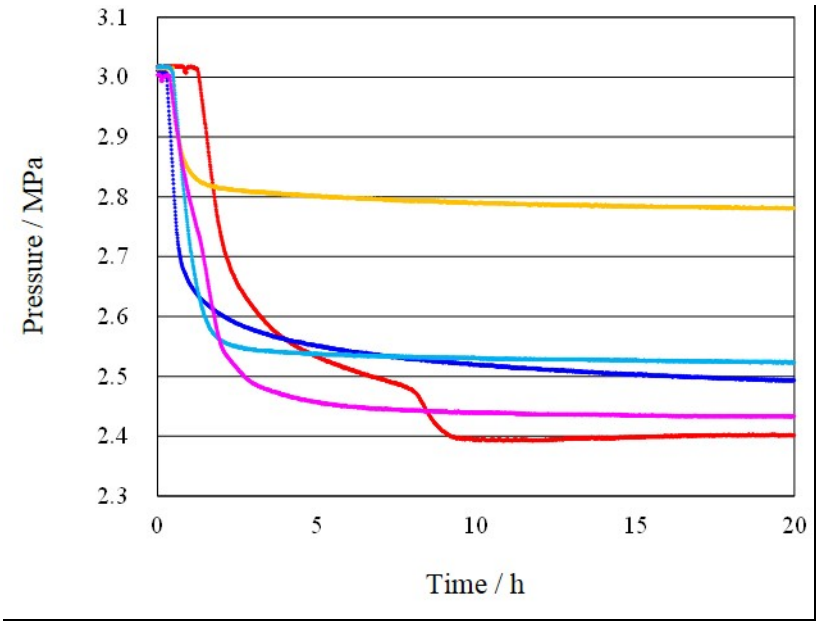

(a) First test

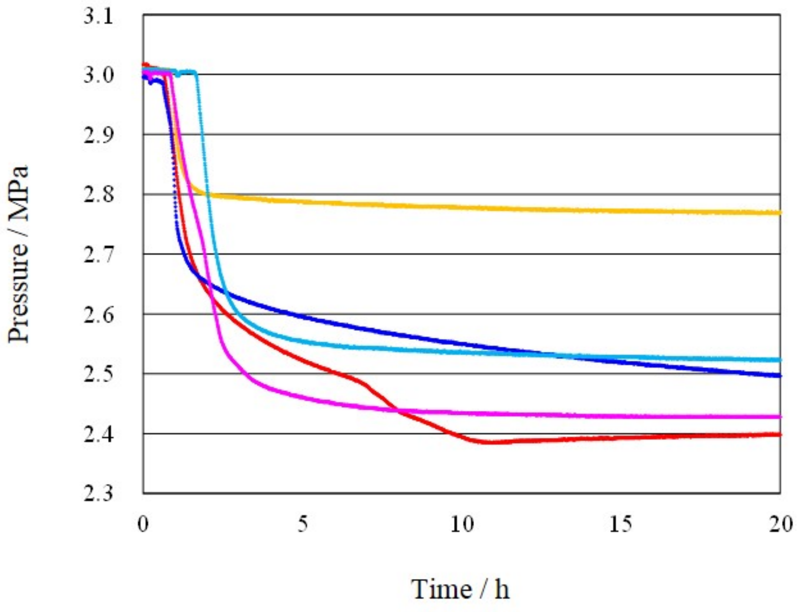

(b) Second test

Figure S4. Pressure trends of gas separation tests at 3 MPa. Colours: red, TBAB with *w* = 0.320; blue, TBAB with *w* = 0.200; orange, TBAC with *w* = 0.200; light blue, TBPB with *w* = 0.200; pink, TBPC with *w* = 0.200

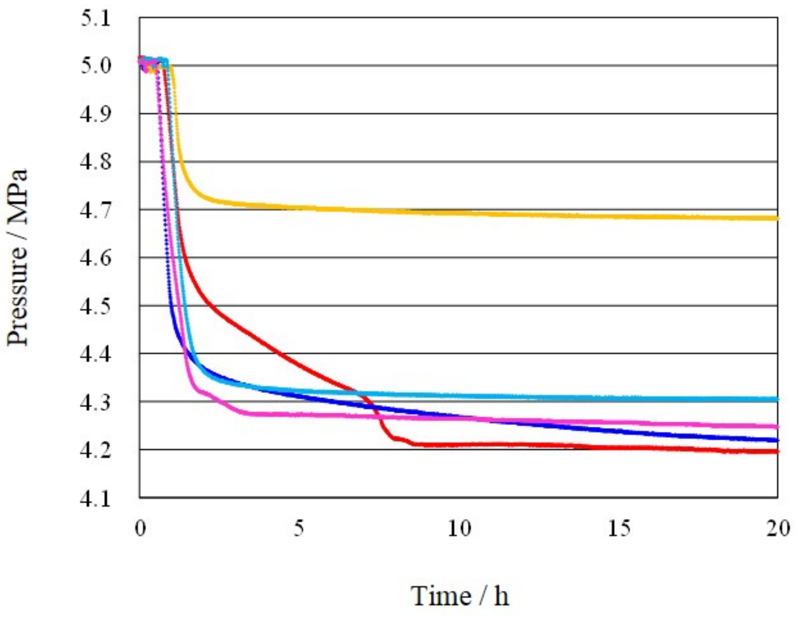

(a) First test

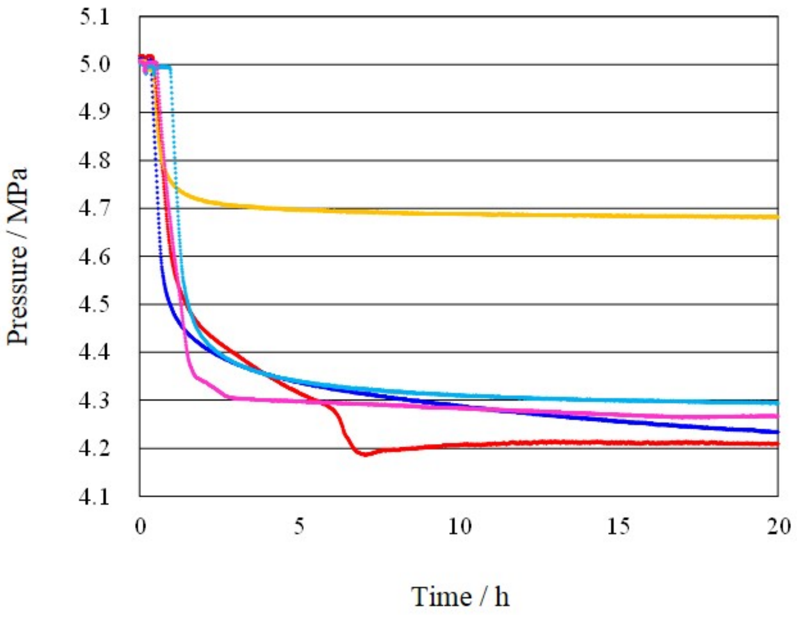

(b) Second test

Figure S5. Pressure trends of gas separation tests at 5 MPa. Colours: red, TBAB with w = 0.320; blue, TBAB with w = 0.200; orange, TBAC with w = 0.200; light blue, TBPB with w = 0.200; pink, TBPC with w = 0.200

Table S1. Composition changes of aqueous solution through gas separation test.

Ionic guest	Hydrate formation	Initial pressure / MPa	Temperature / K	Refractive index	*w*
TBAB	Before	—	298.2	1.386	0.32
w = 0.320	After	3.01	298.2	1.384	0.31
		5.00	298.2	1.386	0.32
TBAB	Before	—	299.2	1.365	0.20
w = 0.200	After	3.00	298.2	1.348	0.10
		5.00	298.2	1.346	0.09
TBAC	Before	—	298.2	1.366	0.21
w = 0.200	After	1.00	298.2	1.352	0.12
		3.01	298.2	1.352	0.12
		5.00	297.2	1.352	0.12
TBPB	Before	—	298.2	1.367	0.20
w = 0.200	After	1.01	298.2	1.354	0.13
		3.01	298.2	1.352	0.12
		5.00	299.2	1.349	0.10
TBPC	Before	—	299.2	1.368	0.20
w = 0.200	After	1.01	299.2	1.357	0.14
		3.00	299.2	1.355	0.12
		5.00	299.2	1.360	0.15

Table S2 summarizes the results of our gas separation tests. Hydrate formation period (t) was shown. T and ΔT_{sub} denote test temperature and subcooling temperature, respectively. P_0 and P_e denote initial pressure and final pressure, respectively. y_{CO_2} and y'_{CO_2} are mole fractions of CO_2 in gas phase before and after hydrate formation, respectively. ϕ_{CO_2}, $n^{H}_{CO_2}$ and n^{H} denote CO_2 composition against N_2 in hydrate phase in mole fraction, amount of CO_2 captured in hydrate phase and total amount of gas (CO_2 + N_2) in hydrate phase, respectively. Separation factor (S.F.) is separation factor defined as follows:

$$S.F. = \frac{n^{G}_{N_2} \times n^{H}_{CO_2}}{n^{G}_{CO_2} \times n^{H}_{N_2}} ,$$

where, $n^{G}_{N_2}$, $n^{G}_{CO_2}$ and $n^{H}_{N_2}$ denote amount of N_2 in gas phase at the end of test, amount of CO_2 in gas phase at the end of test and amount of N_2 captured in hydrate phase, respectively.

Table S2. Experimental conditions and results of the gas separation tests

Guest substance	w mass fraction	t/h	T/K	ΔT_{sub}/K	P_0/MPa	P_e/MPa	y_{CO_2} mole fraction	y'_{CO_2} mole fraction	ϕ_{CO_2} mole fraction	$n^{H}_{CO_2}$/mmol	n^{H}/mmol	*S.F.*
TBAB	0.320	19.0	282.2	3.5	1.02	0.86	0.121	0.081	0.335	11.3	33.8	5.7
		21.2	282.2	3.5	1.02	0.84	0.124	0.080	0.328	12.7	38.8	5.6
		20.4	285.2	2.1	3.02	2.40	0.119	0.075	0.282	38.7	137.1	4.8
		20.4	285.2	2.1	3.02	2.40	0.119	0.076	0.279	38.4	137.6	4.7
		21.2	286.2	2.9	5.02	4.19	0.122	0.089	0.285	53.3	187.2	4.1
		20.3	286.2	2.9	5.02	4.21	0.123	0.088	0.298	55.0	184.5	4.4
TBAB	0.200	20.7	281.2	3.1	1.00	0.79	0.133	0.072	0.358	16.5	46.2	7.2
		21.2	281.2	3.1	1.01	0.80	0.114	0.072	0.272	12.4	45.6	4.8
		24.7	283.2	3.4	3.00	2.48	0.116	0.082	0.274	31.7	115.5	4.2
		23.8	283.2	3.4	3.01	2.49	0.115	0.083	0.261	30.6	117.1	3.9
		24.4	284.2	4.1	5.01	4.21	0.118	0.090	0.258	47.1	182.6	3.5
		24.3	284.2	4.1	5.01	4.22	0.120	0.091	0.268	48.6	181.0	3.7
TBAC	0.200	20.1	283.2	4.0	1.02	0.91	0.111	0.074	0.436	9.8	22.5	9.7
		19.8	283.2	4.0	1.01	0.90	0.118	0.075	0.471	11.1	23.6	11.0
		19.9	285.2	3.1	3.01	2.77	0.116	0.086	0.447	24.2	54.2	8.6

		20.4	285.2	3.1	3.02	2.78	0.116	0.086	0.448	23.9	53.3	8.6
		20.4	286.2	2.5	5.00	4.68	0.125	0.095	0.518	39.8	76.9	10.2
		20.8	286.2	2.5	5.00	4.68	0.123	0.095	0.490	37.6	76.7	9.1
TBPB	0.200	20.3	281.2	1.9	1.01	0.82	0.120	0.083	0.278	11.5	41.3	4.3
		20.2	281.2	1.9	1.02	0.82	0.121	0.085	0.265	11.6	43.7	3.9
		20.5	283.2	2.5	3.01	2.52	0.116	0.086	0.267	29.0	108.5	3.9
		20.0	283.2	2.5	3.02	2.52	0.117	0.088	0.259	28.7	110.7	3.6
		19.9	284.2	3.6	5.02	4.30	0.121	0.087	0.312	51.2	164.0	4.8
		20.0	284.2	3.6	5.00	4.29	0.121	0.089	0.306	49.6	162.0	4.5
TBPC	0.200	20.2	281.2	2.7	1.01	0.81	0.109	0.073	0.252	11.2	44.3	4.3
		19.9	281.2	2.7	1.01	0.81	0.110	0.075	0.252	11.0	43.6	4.2
		19.9	283.2	3.2	3.00	2.43	0.113	0.084	0.233	29.9	128.4	3.3
		20.0	283.2	3.2	3.00	2.43	0.114	0.085	0.235	29.9	127.2	3.3
		20.0	284.2	3.7	5.00	4.27	0.120	0.090	0.286	48.6	169.6	4.0
		19.9	284.2	3.7	5.01	4.25	0.119	0.090	0.268	46.8	174.6	3.7

4. Detailed Crystallographic data for the ionic clathrate hydrates

Detailed crystallographic data for the ionic clathrate hydrates are summarized in Table S3.

Table S3. Crystallographic data for the ionic clathrate hydrates.

	TBAB with w = 0.200	TBAC with w = 0. 200	TBPB with w = 0. 200	TBPC with w = 0. 200
Space group	*Imma*	$P4_2/m$	(Not determined)	*Cmmm*
Lattice	orthorhombic	tetragonal	hexagonal (Possibly orthorhombic)	orthorhombic
Unit cell dimensions	a = 21.419(4) Å<br>b = 25.833(5) Å<br>c = 12.218(2) Å	a = 23.870(3) Å<br>c = 12.497(3) Å	a = 12.0602(17) Å<br>c = 12.585(3) Å	a = 12.036(2) Å<br>b = 21.145(4) Å<br>c = 12.685(3) Å
Crystal size	0.3 mm × 0.3 mm × 0.3 mm	0.2 mm × 0.3 mm × 0.4 mm	0.1 mm × 0.2 mm × 0.3 mm	0.2 mm × 0.2 mm × 0.3 mm
Source	Mo Kα (wave length: 0.71073 Å)			
Index ranges	$-27 < h < 27, -32 < k < 32, -15 < l < 12$	$-30 < h < 30, -30 < k < 30, -16 < l < 13$	$-15 < h < 15, -15 < k < 15, -16 < l < 16$	$-15 < h < 15, -27 < k < 27, -16 < l < 16$
Reflections collected/unique	3939/2333	8402/5081	2446/1470	2081/1380
θ range for data collection min, max	3.154, 27.250	3.035, 27.383	3.237, 27.465	3.212, 27.478
Completeness to 2θ	0.967333	0.984082	0.996188	0.997603
R_{int}/R_{σ}	0.1211/0.0765	0.0758/0.0446	0.1302/0.0427	0.1087/0.0592

5. Single crystals of ionic clathrate hydrate formed under atmospheric pressure of the air

To compare the Raman spectra of the ionic clathrate hydrates formed with or without CO_2 + N_2 gas, we formed the ionic clathrate hydrates in the absence of CO_2 + N_2, i.e., under atmospheric pressure of the air. The obtained crystals were shown in Figure S6.

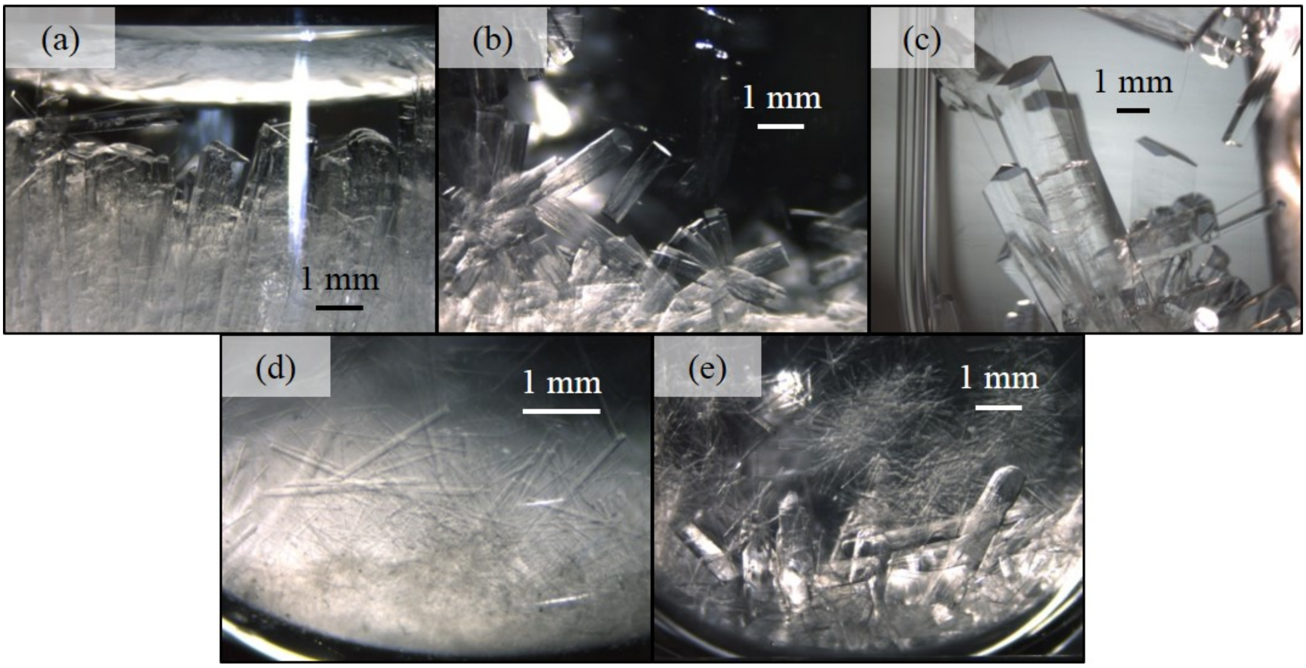

Figure S6. Crystals of ionic clathrate hydrates formed under atmospheric pressure of the air. (a) TBAB hydrates formed with *w* = 0.320 at 280.6 K, (b) TBAB hydrates formed with *w* = 0.200 at 280.6 K, (c) TBAC hydrates formed with *w* = 0.200 at 286.2 K, (d) TBPB hydrates formed with *w* = 0.200 at 280.2 K, (e) TBPC hydrates formed with *w* = 0.200 at 280.2 K.

6. Raman spectra of ionic clathrate hydrate with and without CO_2 + N_2 mixed gas

The presently obtained Raman spectra of ionic clathrate hydrates formed with and without CO_2 + N_2 mixed gas and CO_2 + N_2 + tetrahydrofuran (THF) hydrate are shown in Figure S7. Peaks of C–C–C–C ring breathing modes in THF are observed between 900 and 950 cm^{-1} [S4–S7]. Peaks of C–C stretching vibration modes in TBA^+, TBP^+ [S7,S8] and THF [S7] are observed between 950 and 1400 cm^{-1}. Peaks of C–H bending vibration modes in TBA^+, TBP^+ [S7–S9] and THF [S7] are observed between 1400 and 1500 cm^{-1}. Peaks of O–H stretching vibration modes in water molecules were observed between 3050–3200 cm^{-1} [S5,S10]. Peaks of C–H stretching vibration modes of TBA^+, TBP^+ [S7–S9, S13–S15] and THF[S5–S7] were observed in a range between 2850–3050 cm^{-1}.

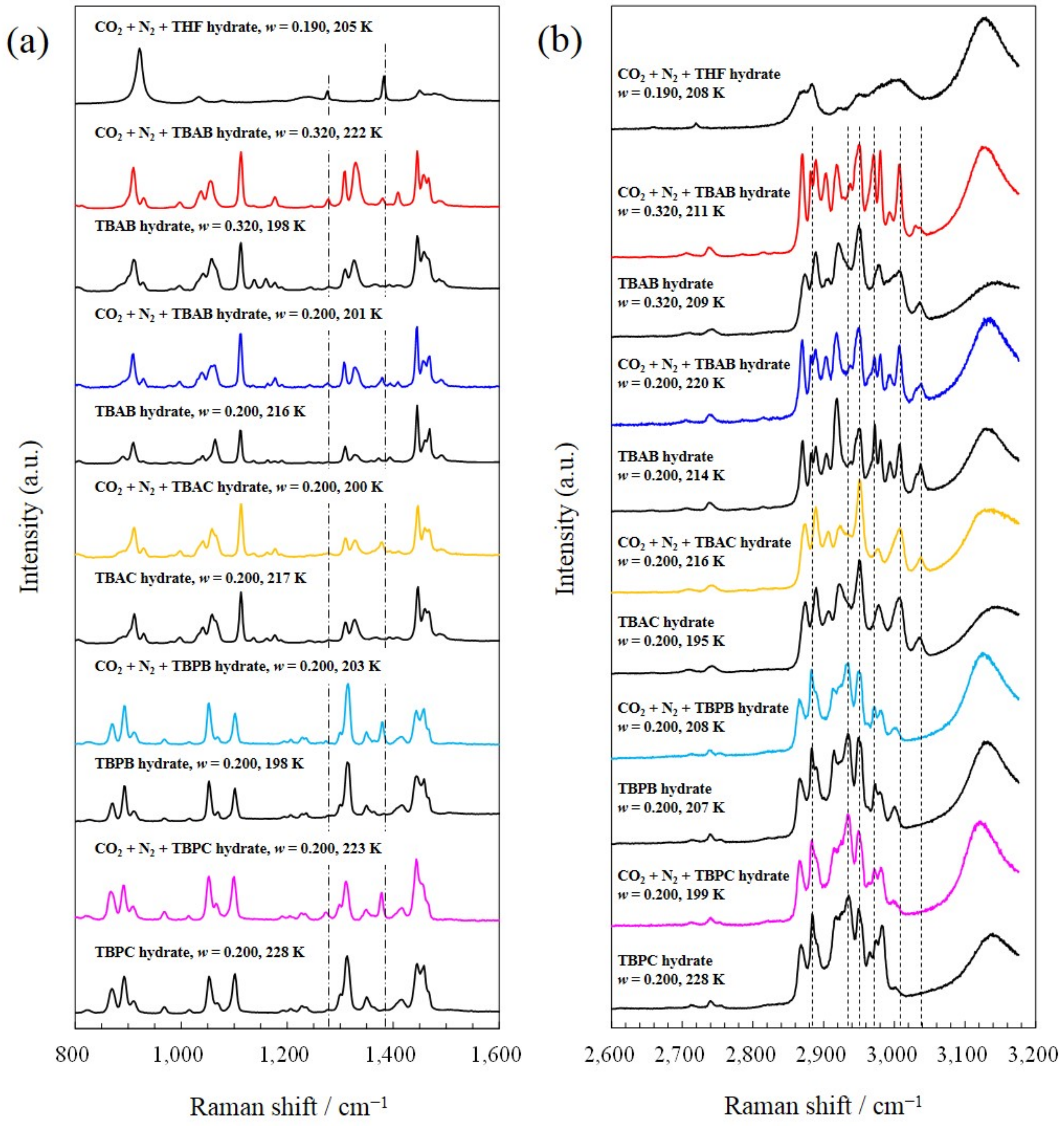

Figure S7. Raman spectra of ionic clathrate hydrates formed in this study. Raman spectra in the wave number region (a) between 800 and 1600 cm^{-1} and (b) between 2600 and 3200 cm^{-1} were obtained with resolution of 1.8 and 0.6 cm^{-1}, respectively. The spectra were analyzed under N_2 atmosphere at 195–228 K. Chain lines indicate CO_2 peaks at 1276 and 1382 cm^{-1}, respectively. Dashed

lines indicate the peaks of C–H stretching vibration modes of TBA^+ or TBP^+ at 2884, 2937, 2947, 2969, 3009 and 3040 cm^{-1}, respectively.

References

S1. Adisasmito, S., Frank, R. J. & Sloan, E. D. Hydrates of carbon dioxide and methane mixtures. *J. Chem. Eng.* Sci. **36**, 68–71 (1991).

S2. Muromachi, S., Hashimoto, H., Maekawa, T., Takeya, S. & Yamamoto, Y. Phase equilibrium and characterization of ionic clathrate hydrates formed with tetra-*n*-butylammonium bromide and nitrogen gas. *Fluid Phase Equilib.* **413**, 249−253 (2016).

S3. Hashimoto, H., Yamaguchi, T., Kinoshita, T. & Muromachi, S. Gas separation of flue gas by tetra-*n*-butylammonium bromide hydrates under moderate pressure conditions. *Energy* **129**, 292–298 (2017).

S4. Hashimoto, S., Murayama, S., Sugahara, T. & Ohgaki, K. Phase equilibria for H_2 + CO_2 + tetrahydrofuran + water mixtures containing gas hydrates. *J. Chem. Eng. Data* **51**, 1884–1886 (2006).

S5. Tulk, C. A., Klug, D. D., & Ripmeester, J. A. Raman spectroscopic studies of THF clathrate hydrate. *J. Phys. Chem. A* **102**, 8734−8739 (1998).

S6. Prasad, P. S. R., Prasad, K. S. & Thakur, N. K. Laser Raman spectroscopy of THF clathrate hydrate in the temperature range 90–300K. *Spectrochim. Acta. A* **68**, 1096–1100 (2007).

S7. Kagel, R. O. Raman Spectroscopy in *Handbook of Spectroscopy Volume II* (ed. Robinson, J. W.) 107–130 (CRC Press, 1974).

S8. Jeghnou, H. *et al*. Structural phase transition in (*n*-$C_4H_9NH_3)_2SiF_6$: DSC and Raman studies. *J. Raman Spectrosc*. **34**, 126–130 (2003).

S9. Jin, Y., Kida, M. & Nagao, J. Phase transition of tetra-*n*-butylammonium bromide hydrates enclosing krypton. *J. Chem. Eng. Data* **61**, 679−685 (2016).

S10. Taylor, M. J. & Whalley, E. Raman spectra of ices Ih, Ic, II, III, and V. *J. Chem. Phys*. **40**, 1660−1664 (1964).

S11. Marckmann, J. P. & Whalley, E. Vibrational spectra of the ices. Raman spectra of ice VI and ice VII. *J. Chem. Phys*. **41**, 1450−1453 (1964).

S12. Ikeda, T., Mae, S. & Uchida, T. Effect of guest–host interaction on Raman spectrum of a CO_2 clathrate hydrate single crystal. *J. Chem. Phys*. **108**, 1352–1359 (1998).

S13. Bator, G. Provoost, R. Silverans, R. E. & Zeegers-Huyskens, Th. Raman study of phase transitions in (*n*-$C_4H_9NH_3)_2BiC1_5$. J. Mol. Struct. **435**, 1–10 (1997).

S14. Jin, Y. & Nagao, J. Change in the stable crystal phase of tetra-*n*-butylammonium bromide (TBAB) hydrates enclosing xenon. *J. Phys. Chem. C* **117**, 6924−6928 (2013).

S15. Chazallon, B., Ziskind, M., Carpentier, Y. & Focsa, C. CO_2 capture using semi-clathrates of quaternary ammonium salt: structure change induced by CO_2 and N_2 enclathration. *J. Phys. Chem. B* **118**, 13440−13452 (2014).

Nomenclature

w	mass fraction of aqueous solution
t	time from hydrate nucleation
T	temperature
ΔT_{sub}	subcooling temperature
P_0	initial pressure
P_e	pressure at the end of test
y_{CO_2}	mole fraction of CO_2 in gas phase before hydrate formation
y'_{CO_2}	mole fraction of CO_2 in gas phase at the end of test
ϕ_{CO_2}	mole fraction of CO_2 in hydrate phase
$n_{CO_2}^{H}$	amount of CO_2 in hydrate phase
n^{H}	total gas amount (CO_2 + N_2) in hydrate phase
S.F.	separation factor
$n_{N_2}^{G}$	amount of N_2 in gas phase at the end of test
$n_{CO_2}^{G}$	amount of CO_2 in gas phase at the end of test
$n_{N_2}^{H}$	amount of N_2 captured in hydrate phase
